# Resilience level and its relationship with hypochondriasis in nurses working in COVID-19 reference hospitals

**DOI:** 10.1186/s12912-021-00730-z

**Published:** 2021-11-02

**Authors:** Ali Reza Yusefi, Salman Daneshi, Esmat Rezabeigi Davarani, Parnian Nikmanesh, Gholamhossein Mehralian, Peivand Bastani

**Affiliations:** 1 Department of Public Health, Healthcare Services Management, School of Health, Jiroft University of Medical Science, Jiroft, Iran; 2 Department of Public Health, Jiroft University of Medical Sciences, Jiroft, Iran; 3 Health in Disasters and Emergencies Research Center, Institute for Future Studies in Health, Kerman University of Medical Sciences, Kerman, Iran; 4 Healthcare Services Management, School of Management and Information Sciences, Iran University of Medical Sciences, Tehran, Iran; 5 Department of Pharmacoeconomics and Pharmacy Administration, School of Pharmacy, Shahid Beheshti University of Medical Sciences, Tehran, Iran; 6 Health Human Resources Research Center, School of Health Management and Information Sciences, Shiraz University of Medical Sciences, Shiraz, Iran

**Keywords:** Resilience, Hypochondriasis, Nurses, Crisis, Coronavirus, COVID-19, Iran

## Abstract

**Introduction:**

A new coronavirus, called COVID-19, is an acute respiratory disease, which may arouse many psychological disorders since there is no specialized knowledge about it. The present study aimed to investigate the level of resilience and its relationship with hypochondriasis in nurses working in a COVID-19 reference hospital in south of Iran.

**Methods:**

This cross-sectional study was conducted in 2020, in which 312 nurses participated using the census method. Data collection tools were the Conker-Davidson standard resilience scale (CD-RISC) and the Evans Hypoglycaemia Awareness Questionnaire. Data were analyzed using t-test, ANOVA, Pearson correlation coefficient, and multiple linear regression using SPSS software version 23.

**Results:**

The mean scores of resilience and hypochondriasis were 72.38 ± 7.11 and 49.75 ± 8.13, respectively, indicating the moderate level of these two variables among nurses. Hypochondriasis in 18.91, 61.22, and 1.28% of the nurses was mild, moderate, and severe, respectively. There was a significant negative correlation between resilience and hypochondriasis (r = − 0.214 and *P* < 0.001). In this regard, control (*P* < 0.001), positive acceptance of change (*P* < 0.001), spiritual effects (*P* = 0.001), trust in individual instincts (*P* = 0.001), and perception of competence (*P* = 0.002) were detected as the predictors of nurses’ hypochondriasis.

**Conclusion:**

The nurses had moderate levels of resilience and hypochondriasis. Promoting knowledge about COVID-19and increasing information on how to protect oneself and others against the disease along with supportive packages from their managers are thus recommended.

## Background

A new and genetically modified virus from the SARS-COV_2 coronavirus family was emerged in December 2019 [1]. The virus spread rapidly worldwide due to its very high transmission power and infected nature in almost all countries in a short time (less than 4 months) [2]. The first case of COVID-19 in Iran was reported on February 19, 2020 [3]. Emerging diseases such as COVID-19 have always been accompanied by high mental load, stress, and anxiety for individuals because of the lack of comprehensive knowledge and awareness about the disease [4]. During the outbreak of COVID-19, nurses, as a first line health profession, have experienced multiple mental disorders, including depression, anxiety, stress, sleep disorders, and aggression, and so on because of the reasons such as particular job positions, high workload, unknown nature of the disease, frequent changes in protocols and operational roles, unprecedented changes in personal plans, rapid policy and information changes, role change, extreme fatigue, exposure to critically ill patients, and high mortality of patients, keeping off from their immediate relatives due to fear of infection, insufficient psychological, social, and organizational support, and lack of personal protective equipment, [5–9].

Under these critical and stressful conditions, one of the main determinants in maintaining individuals’ mental and physical health is their resilience and flexibility. Resilience is defined as individuals’ positive adjustment in response to adverse and difficult situations or their positive adaptation to bitter and unpleasant experiences [10]. The resilient individual faces tensions, challenges, and crises and also actively engages in his personal and work environment [11]. Resilience capacity is considered as a predictive factor to prevent and reduce professional stress [12], and the resilient is an active participant and the constructor of his external environment [13]. Such a person has an acceptable ability to overcome danger and hardship [14] and is resistant to increased mental disorders at the time of danger and hardship [15]. Numerous studies on health workers during the COVID-19 pandemic have documented a significant negative relationship between resilience and job stress, depression, and burnout [16–18].

Resilience is an essential potential for success in nursing activities [19] and corrects or modifies the adverse effects of unfavorable working conditions, enhances mental health, and improves the quality of nursing services [20]. Resilient nurses have a stress-resistant personality and feel that they can find a suitable way to solve their problems in stressful and traumatic conditions in the hospital environment [21]. On the other hand, hypochondriasis disorder is one of the mental disorders which may affect health workers during the COVID-19 outbreak. Hypochondriasis is one of the quasi-physical disorders in which, despite his physical health, a person believes in the existence of disease for at least 6 months [4]. Such individuals may constantly think of the existence of a new disease. Accordingly, this is often associated with anxiety and depression [10]. In the fifth edition of the Diagnostic and Statistical Manual of Mental Disorders (DSM-5), hypochondriasis is reported to be about 2.7%, which is the same in men and women [22], and the highest prevalence is reported in individuals aged 20–30 years [23]. In a study by Loper (2001), the prevalence of hypochondriasis in the general Canadian population was estimated to be 1–2% [24]. In another study, Barsky et al. (1990) estimated the prevalence of hypochondriasis in 136 patients to be between 3.6–4.2% [25]. Studies have revealed that the prevalence of hypochondriasis is higher in health care personnel than in the general population [23, 26, 27].

Among the healthcare staff, nurses who feel a lot of pressure and stress in their work environment and have not been trained to acquire appropriate strategies to deal with such pressures use negative psychological strategies such as hypochondriasis in the face of environmental stress, which may affect their job and social functioning [23]. According to these studies, there is a significant relationship between hypochondriasis with anxiety and depression in nurses [23, 26]. In Iran, after confirming the detection of the first case of COVID-19, about 210 41 referral hospitals and training centers have been provided to track and follow up on suspected and positive cases of the disease [28]. Given that individuals’ and flexibility as well as the feeling of illness (hypochondriasis) in crises such as the COVID-19 pandemic, this study aims to assess the level of resilience and its relationship with hypochondriasis in nurses working in a COVID-19 reference hospital in south of Iran. Findings of this study would provide evidence for policy makers in nursing area as to how deal with unexpected situations and make nurses ready to tackle hypochondriasis and stay resilient in their work environment. In other words, this research help healthcare mangers set some priorities to improve the mental health of nurses as well as their resilience in COVID 19 outbreak.

## Methods

**Fig. 1:**
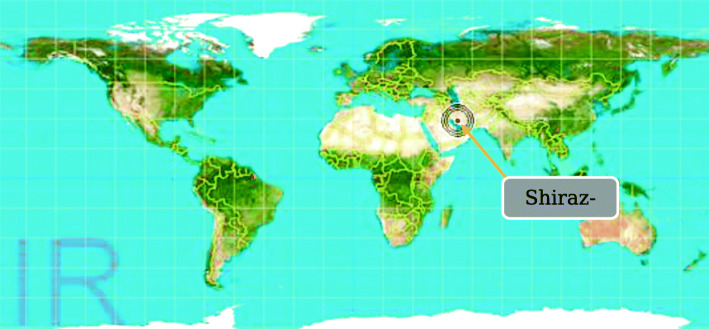
The Geographical Location of the Research. *The map is prepared by one of the authors ARY applying GNU Lesser General Public License 6.2.0 software

This study was a descriptive-analytical cross-sectional study conducted in 2020. The study population consisted of nurses working in Hazrat Ali Asghar (AS) Hospital as the reference hospital of COVID-19 in Fars province, Iran. Figure 1 presents the geographical location of the study.

In this study, the census method was used, and all nurses (*n* = 312) were participated in the study. Upon the nurses willingness to participate in the study and if they were working in clinical wards of the hospital were among the inclusion criteria to enter the study. Working in administrative sectors of the hospital was the main exclusion criteria. The data collection tool was a three-section questionnaire. The first section addressed the nurses’ demographic characteristics (age, gender, work experience type of employment, marital status, and level of education, number of shifts per month, and number of patients monitored per shift). The second section was the Conker-Davidson standard resilience scale (CD-RISC). This scale encompasses 5 subscales and 25 items: perception of competence (8 items), trust in individual instincts (7 items), and positive acceptance of change and secure relationships (5 items), control (3 items), and spiritual effects (2 items). It is scored based on a Likert-scale ranging from one (completely incorrect) and five (completely correct). To determine the nurses’ resilience level, the levels were classified as very favorable (106–125), favorable (86–105), moderate (66–85), unfavorable (46–65), and very unfavorable (25-45). Keyhani et al. examined the psychometric properties of this scale and confirmed its reliability (with Cronbach’s alpha coefficient of 0.66) and validity [29].

The third section was the Evans Hypoglycaemia Awareness Questionnaire (Evans, 1980). This questionnaire has 36 items and measures hypochondriasis based on a Likert scale, according to which individuals were classified in healthy groups (score <16), borderline hypochondriasis (score 16–30), mild hypochondriasis (score 31–45), moderate hypochondriasis (score 46–60) and severe hypochondriasis (score > 60). The validity of the questionnaire was confirmed by the content validity method, and its reliability was confirmed by Cronbach’s alpha coefficient in previous studies [23]. The nurses voluntarily participated in the study and filled out the questionnaires. For research ethics, all questionnaires were anonymous, and all participants have been guided if needed. After obtaining the necessary permits from the Shiraz University of Medical Sciences (SUMS) and explaining the objectives of the project to the participants, the confidentiality principle was emphasized, and their satisfaction was obtained. Then the questionnaires were distributed electronically among the nurses. After completion, the data were imported into SPSS software version 23 and analyzed using descriptive and inferential statistical methods, including T-test, ANOVA, Pearson correlation coefficient, and multiple linear regression at the significant level of 0.05.

## Results

The nurses’ mean age was 31.32 ± 7.18 years, and most of the participants (53.20%) were aged below 30 years old. Their average work experience was 6.24.38 ± 6.38 years, they were mostly in the group with work experience < 10 years (71.47%). In this study, 65.06% were women. Most of the respondents had a bachelor’s degree (88.46%) and contractual employees (58.34%) with 10–20 shifts per month (45.84%). For most of the nurses, more than three patients were monitored in each shift (83.98%) (Table 1).

**Table 1:** Characteristics of Nurses Participating in the Study (*n*_=_ 312)

Variables	Category	Frequency (Percent)
**Age (year)**	< 30	166 (53.20)
	30–40	127 (40.71)
	> 40	19 (6.09)
**Work experience (year)**	< 10	223 (71.47)
	10–20	76 (24.36)
	> 20	13 (4.17)
**Gender**	Man	109 (34.94)
	Woman	203 (65.06)
**Marital status**	Single	69 (22.12)
	Married	243 (77.88)
**Level of education**	Bachelor	276 (88.46)
	Masters	36 (11.54)
**Type of employment**	Official	82 (26.28)
	Temporary-to permanent	7 (2.24)
	Under -a-contract	19 (6.09)
	Contractual	182 (58.34)
	Corporative	22 (7.05)
**Number of shifts per month**	< 10	28 (8.97)
	10–20	143 (45.84)
	> 20	141 (45.19)
**Number of patients monitored in each work shift**	2 patient	7 (2.24)
	3 patient	43 (13.78)
	> 3 patient	262 (83.98)
**Total**	–	**312 (100)**

The nurses’ mean scores of resilience and hypochondriasis were 72.38 ± 7.11 and 49.75 ± 8.13, respectively, indicating moderate levels of these two variables among the nurses. Further, hypochondriasis was mild, moderate, and severe in 18.91, 61.22, and 1.28% of the nurses (Table 2).

**Table 2:** Mean and Standard Deviation of Resilience and Hypochondriasis of studied Nurses

Variables	Dimensions	Score range	Mean ± Std^a^
**Resilience**	Perception of Competence	8–40	22.16 ± 2.14
	Trust in Individual Instincts	7–35	19.87 ± 2.08
	Positive Acceptance of Change	5–25	15.21 ± 1.8
	Control	3–15	9.01 ± 1.62
	Spiritual Effects	2–10	6.13 ± 1.36
	**Total Resilience**	**25–125**	**72.38 ± 7.11**
**Hypochondriasis**	**Domain**	**Frequency**	**Percent**
	Healthy	28	8.97
	Borderline	30	9.62
	Mild	59	18.91
	Moderate	191	61.22
	Severe	4	1.28
	**Total Mean ± Std Hypochondriasis**	**49.75 ± 8.13**	

^a^*Std* Standard Deviation

**Fig. 2:**
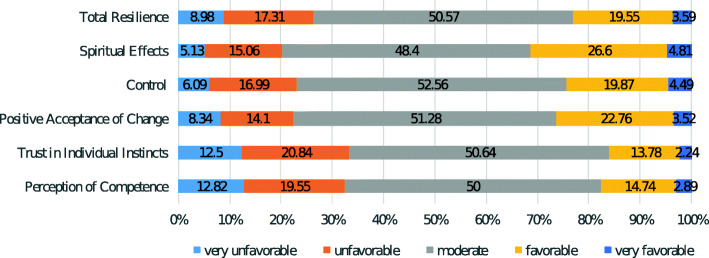
Frequency Distribution of Resilience and its Dimensions among studied Nurses

According to the other results, the resilience level was estimated to be moderate for 50.57% of the nurses. Moreover, among the resilience dimensions, from the perspective of 20.84% ​​of the nurses, the dimension of “trust in individual instincts” was at an unfavorable level (Fig. 2).

The Kolmogorov-Smirnov test indicated that the data are normally distributed. The results showed a significant negative correlation between resilience and its dimensions with hypochondriasis in the concerned nurses (r = − 0.214 and *P* < 0.001). Among the resilience dimensions, “control” had the highest correlation with hypochondriasis (r = − 0.221 and *P* < 0.001) (Table 3).

**Table 3:** Correlation between Resilience and Hypochondriasis of Nurses Participating in the Study

	Dimensions of Resilience					Total Resilience
	Perception of Competence	Trust in Individual Instincts	Positive Acceptance of Change	Control	Spiritual Effects	
**Total** **Hypochondriasis** r(p)^a^	- 0.198 (0.001)	- 0.209 (0.001)	- 0.216 (< 0.001)	- 0.221 (< 0.001)	- 0.212 (0.001)	- 0.214 (< 0.001)

^a^*r* Pearson Correlation Coefficient, *P P*-Value (Correlation is significant at the 0.05 level)

The results of multiple linear regression analysis were used to determine the effect of different resilience dimensions and demographic characteristics (with a significant relationship) on the hypochondriasis in the studied nurses and showed that the significant variables in the model, which were determined using the Enter method, were “control”, “positive acceptance of change”, “spiritual effects”, and “trust in individual instincts”, “perception of competence”, “number of patients monitored in each work shift”, “number of shifts per month”, “level of education”, and “gender”, respectively. This test also showed that the coefficient of determination for the processed model (R-Adjusted) was 0.53, indicating that 53% of the variation in the hypochondriasis score can be explained by the model variables. According to the multiple linear regression analysis of the linear equation, the nurses’ hypochondriasis scores were calculated as follows:

$$ {\mathrm{Y}}_{=}0.611-0.366 \times_1-0.347 \times_2-0.278 \times_3-0.241 \times_4-0.211 \times_{5+}0.189\ {\times}_{6+}0.174 \times_{7+}0.131\ {\times}_{8+}+0.119\ {\times}_9 $$

Where, Y is hypochondriasis score and x_1–9_ are variables affecting hypochondriasis in nurses (Table 4).

**Table 4:** Factors Affecting Hypochondriasis using Multiple Linear Regression Model

Variable definition	Variable	Unstandardized coefficients		Standardized coefficient β	***P***-value*
		B*	Std. Error*		
**–**	(Constant)	0.611	1.46	–	0.003
**x**_**1**_	Control	- 0.366	0.058	- 0.342	< 0.001
**x**_**2**_	Positive Acceptance of Change	- 0.347	0.067	- 0.321	< 0.001
**x**_**3**_	Spiritual Effects	- 0.278	0.072	- 0.267	0.001
**x**_**4**_	Trust in Individual Instincts	- 0.241	0.076	- 0.219	0.001
**x**_**5**_	Perception of competence	- 0.211	0.091	- 0.198	0.002
**x**_**6**_	Number of patients monitored in each work shift	0.189	0.083	0.175	0.002
**x**_**7**_	Number of shifts per month	0.174	0.088	0.163	0.004
**x**_**8**_	Level of education	0.131	0.091	0.154	0.021
**x**_**9**_	Gender	0.119	0.11	0.101	0.032

^*^P-value Correlation is significant at the 0.05 level, *B* Unstandardized coefficients, *Std. Error* Standard Error

According to the results, there was a statistically significant relationship between the mean score of resilience with age (*p* = 0.006), work experience (*p* = 0.02), number of shifts per month (*p* = 0.04), and number of patients under observation (*p* = 0.002) and the mean score of hypochondriasis with gender (*p* = 0.04), level of education (*p* = 0.03), number of shifts per month (*p* = 0.002), and patient under observation (*p* = 0.001). In addition, according to post-hoc test, nurses with more than 20 work shifts per month and responsibility to care three patients in each work shift had less resilience and higher hypochondriasis compared to others (Table 5).

**Table 5:** Relationship between Variables of Resilience and Hypochondriasis with Demographic Characteristics of Nurses

Variables	Demographic Variables						Number of shifts	Number of patients
	Age	Work experience	Gender	Marital status	Type of employment	Level of education		
**Resilience**	r =* 0.301 *P* =* 0.006	r = 0.211 *P* = 0.02	t =* 1.861 *P* = 0.11	t = 1.632 *P* = 0.09	F =* 1.229 *P* = 0.26	t = 1.367 *P* = 0.16	F = 2.118 *P* = 0.04	F = 3.081 *P* = 0.002
**Hypochondriasis**	r = 0.138 *P* = 0.10	r = 0.144 *P* = 0.09	t = 1.522 *P* = 0.04	t = 1.611 *P* = 0.07	F = 1.563 *P* = 0.13	t = 2.342 *P* = 0.03	F = 3.112 *P* = 0.002	F = 3.271 *P* = 0.001

^*^r Pearson Correlation Coefficient, *P P*-Value, *t* T-Test, *F* Test ANOVA, (Correlation is significant at the 0.05 level)

## Discussion

The present study was to determine the level of resilience and its relationship with hypochondriasis among the nurses working in a COVID-19 reference hospital in south of Iran. According to the findings, the nurses’ resilience level was moderate. Consistent with this finding, the findings of some other studies conducted during the COVID-19 pandemic indicated moderate levels of resilience among nurses [17, 30, 31]. Moreover, results of Roberts’ et al. (2021) during the COVID-19 pandemic in the UK showed that the nurses’ resilience score of 65% was moderate [32]. In another study by Luceño-Moreno et al. (2020) in Spain during the COVID-19 pandemic, moderate levels of resilience were reported among health personnel [9]. Resilience is a complex and dynamic process, which is not only affected by profession but also by various factors such as personal characteristics and environmental and social factors [32, 33].

Regarding the findings of the present study, the nurses’ level of hypochondriasis was also moderate, and 81.41% of the nurses experiences some degrees of hypochondriasis from mild to severe. According to Khani et al. (2016), 45.4% of nurses reported some degrees of hypochondriasis [23]. Furthermore, in Akhavan’s et al. (2019) study, the prevalence of hypochondriasis among the nurses working in the operating room was 18.19%, and unlike the present study, hypochondriasis was mostly at a normal or borderline level [27]. One of the reasons for the high level of hypochondriasis in nurses in the present study, compared to the previous two studies, can be the mental health disorders caused by the COVID-19 pandemic. A study by Kim et al. (2021) revealed that nurses in the COVID-19 wards suffered more from mental health disorders than the other hospital staff [34].

Many problems and challenges experienced by nurses during the COVID-19 pandemic seem to have increased the level of hypochondriasis. In a study conducted during the COVID-19 pandemic on samples other than health personnel, the mean score of hypochondriasis was 33.37 [35], which was lower than the score obtained by the nurses in the present study and higher than those reported in some studies before the COVID-19 outbreak. This could represent the specific conditions posed by the crisis and the impact of the COVID-19 pandemic on all members of society. The prevalence of COVID-19 is such that it has caused psychological consequences such as autoimmune disorder as one of the psychological consequences of epidemic diseases [36]. This disorder can lead to high levels of distress, sadness, depression, and anxiety, as well as decreased levels of useful activities [37].

The findings of the present study showed a significant negative correlation between resilience and its dimensions with hypochondriasis among the studied nurses, suggesting that along with increasing resilience, the nurses’ hypochondriasis decreased.

According to the findings of a study during the COVID-19 pandemic, hypochondriasis significantly increased job stress level [38]. The findings of studies by Mousavi et al. (2019) and Yazdanirad et al. (2021) revealed a significant negative relationship between hypochondriasis and resilience during the COVID-19 pandemic [35, 38]. Studies during the COVID-19 pandemic have suggested a relationship between other mental disorders and resilience among nurses and other health care workers [17, 32].

According to the findings of the present study, the resilience dimensions (control, acceptance of change, spiritual influence, trust in instincts, and individual competence) were identified as predictors of nurses’ hypochondriasis. In Kim’s et al. (2021) study during the COVID-19 pandemic, resilience and spirituality were two strong predictors of mental health problems in nurses, and nurses with high levels of resilience and spirituality were two to six times less likely to suffer from mental disorders [34]. Furthermore, Zhang et al. (2020) reported that those with higher levels of spiritual strength were less likely to experience mental problems during the COVID-19 pandemic [39].

The findings of the present study indicated a significant positive correlation between the mean score of resilience with age and work experience. Accordingly, with increasing age and work experience among nurses, resilience increased. This finding was in line with those reported by Afshari et al. (2021), Kim et al. (2021), Sul et al. (2015), Ang et al. (2018), Lee. Et al. (2015), Ansari Shahidi et al. (2018) and Geraminejad et al. (2018) [31, 34, 40–44]. Thus, older nurses with more work experience seem to have a higher ability to cope successfully with these critical situations because of their more practical experience in difficult situations similar to the COVID-19 pandemic.

In this study, a statistically significant relationship was observed between the mean score of resilience and hypochondriasis with the number of shifts per month and the number of patients under observation. Nurses with more than 20 shifts per month and more than three patients under their direct care in each shift experienced lower levels of resilience and higher levels of hypochondriasis. Khani’s et al. (2016) results showed a statistically significant relationship between the number of nurses’ shifts per month and depression and also between depression and hypochondriasis [23]. Some other studies have suggested that nurses’ relaxation and mental health decrease with increasing work-related fatigue [45–47]. Covering a large number of shifts makes nurses be away from their families and bear the further workload. Under such a condition, nurses are more prone to mental and emotional disorders, and, consequently, psychological problems will play a role as the cause of hypochondriasis. Furthermore, with an increase in the number of patients under care in each shift and regarding the higher workload and higher levels of physical and mental stress posed on nurses, this issue can arouse cognitive disorders such as hypochondriasis in this group.

The findings also revealed a significant difference in the mean scores of hypochondriasis between men and women, indicating that hypochondriasis was higher in women. The finding was in line with those reported by Talaei et al. (2009) and Kim et al. (2021) [34, 48]. The female nurses’ responsibilities of raising children and managing the household, along with the job responsibilities of female nurses, which have accompanied the COVID-19 pandemic as an occupational stressor, exposes this group to a greater risk of mental health disorders such as hypochondriasis.

Finally, a statistically significant relationship was observed between the mean score of hypochondriasis and level of education, suggesting that hypochondriasis was higher in nurses with a bachelor’s degree than in nurses with a master’s degree. In Arasteh’s et al. (2008) study, the prevalence of mental disorders decreased with increasing level of education, so that the highest rate of mental health disorders was noticed in nurses, while the lowest rate was observed in physicians [49]. Moreover, Talaei et al. (2009) and Abolhassani et al. (2014) claimed that hypochondriasis was higher in the group with lower levels of education [48, 50]. Therefore, a higher level of education as an effective factor in improving nurses’ job positions can be effective in promoting their mental health. At the same time, those with higher degrees may fulfill higher ranks the same as managerial positions of the wars and as a result less direct encountering with the patients and treatment procedures. Further, higher income levels in nurses with higher levels of education may make them feel more secure and reduce their stress.

## Conclusion

The nurses had moderate levels of resilience and hypochondriasis. In the outbreak of coronavirus, which may affect nurses’ levels of resilience and hypochondriasis, promoting knowledge about COVID-19 and increasing information on how to protect oneself and others against the disease along with supportive packages from their managers are recommended to be on the policymakers’ agenda and senior university administrators. The main limitation of the present study was its cross-sectional nature, which limited the generalization of the findings; hence, future studies are recommended to be conducted longitudinally and comparatively. Working on a large sample is one of the main strength in this study. As a weaknesse, for future research, it is suggested to consider the ward where nurses work as this may affect their level of resilience and hypochondriasis. It is also recommended to consider the frame of this study to do further research on other health professions such as physicians, physical therapists, paramedics, pharmacists.

## Data Availability

All the data is presented as a part of tables or figures. Additional data can be requested from the corresponding author.

## Abbreviations

CD-RISC: Conker-Davidson standard resilience scale
DSM-5: Diagnostic and Statistical Manual of Mental Disorders
